# Acute Respiratory Distress Syndrome as a Rare but Potentially Fatal Complication of Acute Type B Aortic Dissection: A Case Report

**DOI:** 10.7759/cureus.111243

**Published:** 2026-06-21

**Authors:** Hafsa Erregui, Mehdi Moujahid, Hamza Madouim, Abdelhafid Houba, Nawfal Doghmi

**Affiliations:** 1 Cardiology, Mohammed V Military Teaching Hospital, Rabat, MAR; 2 Cardiology, Faculty of Medicine and Pharmacy of Rabat, Rabat, MAR; 3 Intensive Care Medicine, Mohammed V Military Teaching Hospital, Rabat, MAR

**Keywords:** ards, elderly, hemodialysis, non-cardiogenic pulmonary edema, systemic inflammation, type b aortic dissection

## Abstract

Acute respiratory distress syndrome (ARDS) is an exceptionally rare complication of acute Stanford type B aortic dissection. We report the case of an 84-year-old woman with hypertension and end-stage renal disease on chronic hemodialysis who presented with acute interscapular and chest pain. Computed tomography angiography confirmed an uncomplicated Stanford type B aortic dissection without rupture, malperfusion syndrome, or other indications for urgent intervention. The patient was initially hemodynamically stable and managed conservatively according to current guideline recommendations. Approximately 24 hours after admission, she developed rapidly progressive dyspnea and severe hypoxemic respiratory failure. Chest imaging demonstrated bilateral pulmonary infiltrates, while cardiogenic, embolic, and infectious causes were systematically excluded. Arterial blood gas analysis revealed a partial pressure of arterial oxygen (PaO₂)/fraction of inspired oxygen (FiO₂) ratio of 67 mmHg, fulfilling the Berlin criteria for severe ARDS. Despite invasive mechanical ventilation, prone positioning, and maximal supportive care, respiratory failure progressed rapidly, resulting in multiorgan failure and death approximately 32 hours after admission. This case highlights a rare but potentially fatal inflammatory complication of acute type B aortic dissection. The temporal association, marked inflammatory response, and exclusion of alternative etiologies support the hypothesis of inflammation-mediated lung injury. Early recognition of unexplained respiratory deterioration may facilitate closer monitoring and timely supportive management, particularly in high-risk patients with advanced age and chronic kidney disease.

## Introduction

Acute aortic dissection is a life-threatening vascular emergency associated with substantial morbidity and mortality. Stanford type B aortic dissection, involving the descending thoracic aorta without ascending aortic involvement, accounts for approximately 25-40% of all acute aortic dissections [1]. Current management is primarily guided by the presence or absence of complications, with optimal medical therapy remaining the cornerstone of treatment for uncomplicated cases according to the 2022 American College of Cardiology/American Heart Association (ACC/AHA) Guideline for the Diagnosis and Management of Aortic Disease [2].

Although the classical complications of acute type B aortic dissection include aortic rupture, malperfusion syndromes, refractory pain, and uncontrolled hypertension, systemic inflammatory manifestations have increasingly been recognized. Acute respiratory distress syndrome (ARDS) is a severe form of inflammatory lung injury characterized by acute-onset respiratory failure, bilateral pulmonary infiltrates, non-cardiogenic pulmonary edema, and impaired oxygenation according to the Berlin definition. However, ARDS remains an exceptionally uncommon complication of acute type B aortic dissection, with only a limited number of cases reported in the literature to date [3-6].

The pathophysiological mechanisms linking acute aortic dissection and ARDS are not fully understood but are thought to involve a systemic inflammatory response triggered by extensive aortic wall injury, cytokine release, endothelial activation, and increased vascular permeability. Elevated concentrations of inflammatory mediators, particularly interleukin (IL)-6 and C-reactive protein (CRP), have been associated with disease severity and adverse clinical outcomes in acute aortic syndromes [7-9]. These mechanisms may be particularly relevant in patients with end-stage renal disease (ESRD), a condition associated with chronic inflammation, immune dysregulation, oxidative stress, endothelial dysfunction, and impaired clearance of inflammatory mediators [10,11]. Consequently, patients undergoing chronic hemodialysis may be especially vulnerable to exaggerated inflammatory responses and acute lung injury during major vascular events.

We report the case of an 84-year-old woman undergoing chronic hemodialysis who presented with an initially uncomplicated Stanford type B aortic dissection and subsequently developed fulminant ARDS leading to fatal multiorgan failure. This case highlights a rare but devastating inflammatory complication of aortic dissection and underscores the importance of recognizing unexplained respiratory deterioration, rising inflammatory markers, and new pulmonary infiltrates in patients whose initial presentation may otherwise appear clinically stable.

## Case presentation

An 84-year-old woman with a history of hypertension, ESRD on chronic hemodialysis (three sessions weekly), hypothyroidism, epilepsy, Parkinsonism, and cognitive impairment presented to the emergency department with acute-onset interscapular and chest pain of approximately 12 hours' duration. She had no history of chronic pulmonary disease, recent infection, or known inflammatory disorder.

On admission, the patient was conscious, cooperative, and hemodynamically stable, although markedly hypertensive, with a blood pressure of 180/70 mmHg. Heart rate was 72 beats/minute, within the normal range. Cardiovascular examination revealed no signs of hemodynamic compromise or peripheral hypoperfusion. Cardiac auscultation was unremarkable, with no audible murmurs, gallop rhythm, or pericardial friction rub. Peripheral pulses were symmetrical and intact, with no evidence of pulse deficit.

Initial investigations, including electrocardiography, showed no evidence of acute ischemia or arrhythmia. Bedside transthoracic echocardiography demonstrated a non-dilated left ventricle with preserved systolic function, no regional wall motion abnormalities, no significant valvular disease, and no pericardial effusion. The right ventricle was of normal size and function, with no signs of pulmonary hypertension, thereby excluding a primary cardiogenic etiology (Figure 1).

**Figure 1:**
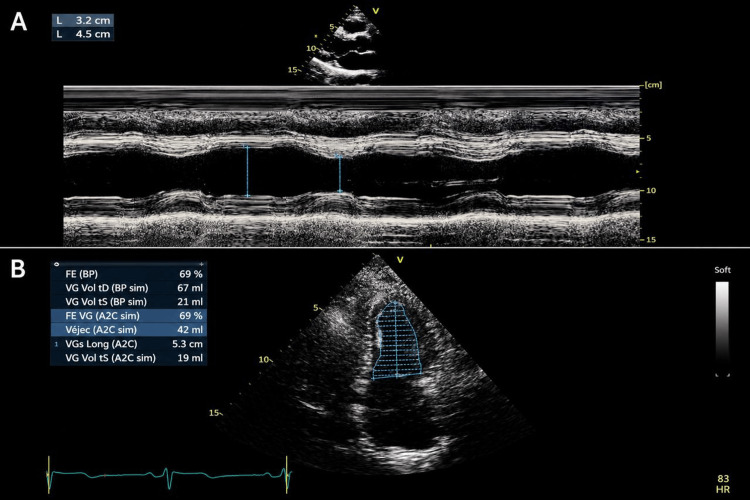
Transthoracic echocardiography demonstrating a non-dilated left ventricle with preserved systolic function. (A) M-mode parasternal long-axis view showing normal left ventricular dimensions; (B) Apical two-chamber view with Simpson biplane analysis demonstrating preserved left ventricular ejection fraction (69%). FE: *Fraction d'Éjection* (ejection fraction); VG: *Ventriculaire Gauche* (left ventricular)

In view of the clinical suspicion of an acute aortic syndrome, contrast-enhanced computed tomography angiography (CTA) was promptly performed, confirming an acute, uncomplicated Stanford type B aortic dissection involving the descending thoracic aorta. A visible intimal flap separated the true and false lumens (Figures 2A-2B). Importantly, there was no evidence of aortic rupture, periaortic hematoma, branch-vessel malperfusion syndrome, significant pleural effusion, pulmonary embolism, or rapid aortic expansion, supporting an initial conservative management strategy according to contemporary guidelines. Notably, lung-window reconstruction obtained during the same examination demonstrated a focal right-sided ground-glass opacity and subtle inflammatory parenchymal changes (Figure 2C). Although non-specific and insufficient to establish a diagnosis of ARDS at presentation, these findings may have represented early inflammatory lung involvement preceding the subsequent respiratory deterioration.

**Figure 2:**
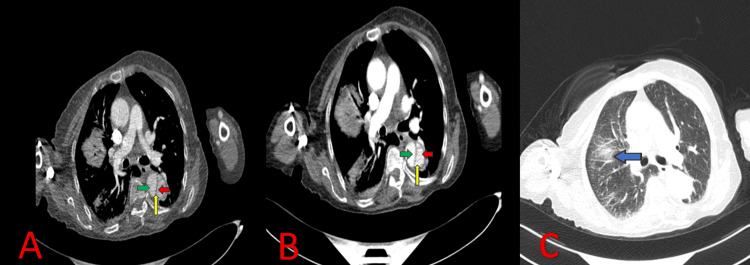
CTA findings at admission demonstrating acute Stanford type B aortic dissection and early pulmonary abnormalities. (A) Cranial axial section and (B) caudal axial section demonstrating an acute Stanford type B aortic dissection involving the descending thoracic aorta. The intimal flap (yellow arrow) separates the true lumen (green arrow) from the false lumen (red arrow). No evidence of aortic rupture, periaortic hematoma, or significant pleural effusion is present. (C) Lung-window reconstruction obtained during the same examination showing a focal right-sided ground-glass opacity (blue arrow). Although non-specific and insufficient to establish a diagnosis of ARDS at presentation, this finding may represent an early inflammatory pulmonary abnormality that preceded the subsequent development of diffuse bilateral infiltrates and severe ARDS. ARDS: acute respiratory distress syndrome; CTA: computed tomography angiography

The patient was admitted to the cardiac intensive care unit and managed conservatively in accordance with contemporary guideline recommendations for uncomplicated Stanford type B aortic dissection. Intravenous labetalol and nicardipine were administered to achieve strict heart rate and blood pressure control, along with adequate analgesia and close hemodynamic monitoring. In the absence of aortic rupture, malperfusion syndrome, refractory pain, uncontrolled hypertension, or rapid aortic expansion, no indication for urgent thoracic endovascular aortic repair (TEVAR) was identified.

Approximately 24 hours after admission, the patient experienced abrupt respiratory deterioration characterized by progressive dyspnea, worsening hypoxemia, and increasing oxygen requirements. Lung-window reconstruction from the initial thoracic CT scan demonstrated patchy ground-glass opacities and early inflammatory parenchymal changes (Figure 2C). Given the patient's rapidly worsening respiratory status and subsequent hemodynamic instability, repeat thoracic CT imaging was not feasible. Follow-up assessment therefore relied on serial chest radiographs, which demonstrated progression from unilateral pulmonary infiltrates to diffuse bilateral opacities consistent with severe ARDS (Figure 3).

**Figure 3:**
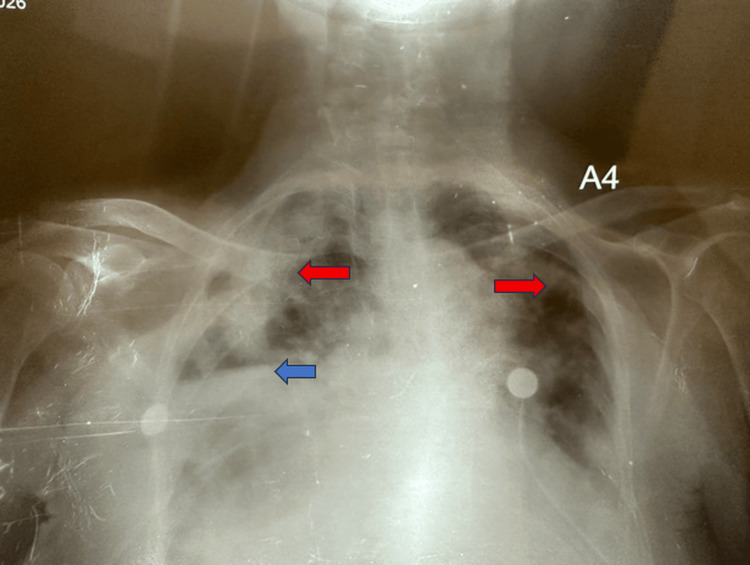
Chest radiograph demonstrating bilateral diffuse alveolo-interstitial opacities (red arrows) with areas of more confluent basal consolidation (blue arrow), consistent with diffuse lung injury in the appropriate clinical context.

Given the abrupt respiratory deterioration, a comprehensive diagnostic workup was undertaken to elucidate the etiology of the acute respiratory failure and systematically exclude cardiogenic, embolic, and infectious causes. A repeat electrocardiogram showed no ischemic changes, and serial high-sensitivity cardiac troponin measurements remained negative. Repeat N-terminal pro-B-type natriuretic peptide (NT-proBNP) testing showed no significant increase compared with baseline values. Although interpretation of natriuretic peptide levels is limited in patients undergoing chronic hemodialysis because of chronically elevated baseline concentrations, the absence of a significant rise, together with negative cardiac biomarkers and preserved left ventricular systolic function on repeat echocardiography, argued against acute cardiogenic pulmonary edema. Pulmonary embolism had previously been excluded by CTA, while no clinical, microbiological, or biological findings supported a primary infectious origin. Blood cultures remained negative, and a low procalcitonin level (0.04 ng/mL) further argued against bacterial infection.

Arterial blood gas (ABG) analysis, obtained shortly after initiation of mechanical ventilation, revealed severe gas exchange abnormalities, with a pH of 7.01 (reference range: 7.35-7.45), partial pressure of carbon dioxide (PaCO₂) >130 mmHg (reference range: 35-45 mmHg), partial pressure of oxygen (PaO₂) of 67 mmHg (reference range: 80-100 mmHg), and lactate of 1.64 mmol/L (reference range: 0.5-2 mmol/L), consistent with profound ventilatory failure and severe respiratory acidosis despite mechanical ventilation. Laboratory investigations demonstrated markedly elevated inflammatory markers, including CRP of 200 mg/L (reference range: <5 mg/L) and leukocytosis of 15,000/mm³ (reference range: 4,000-10,000/mm³), in the presence of a low procalcitonin level of 0.04 ng/mL (reference range: <0.05 ng/mL), suggesting a systemic inflammatory response rather than bacterial infection. Renal function remained severely impaired, with a serum creatinine level of 40 mg/L and an estimated glomerular filtration rate of 7 mL/min/1.73 m², consistent with ESRD requiring chronic hemodialysis.

Despite escalation of oxygen therapy, respiratory failure progressed rapidly, requiring endotracheal intubation and invasive mechanical ventilation. The profound hypercapnia and severe respiratory acidosis reflected advanced ventilatory failure in the setting of rapidly progressive acute lung injury and severe impairment of gas exchange. No clinical or radiological evidence of concomitant obstructive airway disease, mucus plugging, or other alternative explanation for the marked carbon dioxide retention was identified. These findings underscored the severity of the respiratory compromise despite the prompt initiation of invasive ventilatory support.

The diagnosis of ARDS was established according to the Berlin definition [3]. Respiratory symptoms developed within one week of a recognized clinical insult, chest imaging demonstrated bilateral pulmonary infiltrates (Figure 3), and cardiogenic pulmonary edema was excluded by repeat echocardiography, stable NT-proBNP levels, and negative cardiac biomarkers. ABG analysis revealed a PaO₂ of 67 mmHg while receiving an FiO₂ of 1.0, corresponding to a PaO₂/FiO₂ ratio of 67 mmHg and fulfilling the criteria for severe ARDS. Pulmonary embolism and infection were also excluded. Given the close temporal relationship between the onset of respiratory failure and acute type B aortic dissection, together with the marked inflammatory response, a systemic inflammatory response secondary to the underlying aortic injury was considered the most likely precipitating mechanism.

Supportive management included lung-protective mechanical ventilation and prone positioning. Hemodialysis-related fluid management was closely coordinated with intensive care monitoring in an effort to balance the competing challenges of severe ARDS and acute aortic dissection. Despite maximal supportive treatment, including invasive ventilatory support, respiratory failure progressed rapidly, culminating in refractory hypoxemia and multiorgan failure. Given the patient's advanced age, substantial comorbidity burden, and rapidly deteriorating clinical condition, the prognosis remained extremely poor. The patient died approximately 32 hours after admission and nearly eight hours after the onset of severe respiratory deterioration.

The sequence of major clinical events from symptom onset to death is summarized in Table 1.

**Table 1:** Timeline of major major clinical events, diagnostic investigations, therapeutic interventions, and outcome from symptom onset to death. ARDS: acute respiratory distress syndrome; CTA: computed tomography angiography; ECG: electrocardiogram; FiO₂: fraction of inspired oxygen; PaO₂: partial pressure of oxygen; TTE: transthoracic echocardiography; LVEF: left ventricular ejection fraction

Time Point	Event	Key Findings
Symptom onset (0 hours)	Initial presentation	Sudden interscapular and chest pain
~12 hours after symptom onset	Hospital admission	Hemodynamically stable; no respiratory symptoms
Admission	Diagnostic evaluation	TTE demonstrated preserved LVEF without cardiogenic pulmonary evidence; CTA confirmed uncomplicated Stanford type B aortic dissection
	Initial management	Intravenous labetalol and nicardipine; conservative management according to guideline recommendations
~24 hours after admission	Respiratory deterioration	Progressive dyspnea, worsening hypoxemia, and increasing oxygen requirements
	Diagnostic reassessment	Repeat ECG and serial cardiac troponins negative; pulmonary embolism excluded by CTA; infectious workup negative; bilateral pulmonary infiltrates on chest imaging
~ 36 hours after admission	Severe ARDS and death	PaO₂/FiO₂ = 67 mmHg; endotracheal intubation, lung-protective mechanical ventilation, and prone positioning; Progressive multiorgan failure followed by death

## Discussion

The present case is noteworthy because the initial clinical presentation was entirely consistent with an uncomplicated Stanford type B aortic dissection, a condition that is generally managed successfully with optimal medical therapy. At admission, there was no evidence of aortic rupture, branch-vessel malperfusion, refractory pain, uncontrolled hypertension, significant pleural effusion, pulmonary embolism, or rapid aortic expansion. Conservative management was therefore fully aligned with contemporary guideline recommendations [2]. Contrary to the expected clinical course, however, the patient subsequently developed severe ARDS and rapidly progressive multiorgan failure despite initial hemodynamic stability. This discrepancy between the initially favorable presentation and the subsequent fulminant deterioration constitutes the principal educational message of this report.

ARDS complicating acute type B aortic dissection remains exceptionally uncommon and poorly documented. To better contextualize our findings, we reviewed previously reported cases of ARDS associated with Stanford type B aortic dissection (Table 2). Only a limited number of cases have been reported in the literature [4-6]. Litmathe and Dapunt described severe ARDS requiring extracorporeal membrane oxygenation (ECMO) support in a patient with traumatic type B aortic dissection [4]. Xuan et al. reported intraoperative ARDS in a patient with an uncomplicated type B aortic dissection undergoing laparoscopic pancreaticoduodenectomy, with a favorable outcome following supportive management [5]. More recently, Miyagawa et al. described successful treatment of severe ARDS secondary to acute type B aortic dissection using venovenous ECMO and continuous renal replacement therapy [6]. Compared with these previously reported patients, our patient was substantially older, had ESRD requiring chronic hemodialysis, and experienced a much more rapid progression to multiorgan failure and death. These observations highlight the rarity of this complication and suggest that advanced age and chronic renal disease may contribute to an unfavorable prognosis.

**Table 2:** Previously reported cases of ARDS associated with Stanford type B aortic dissection ARDS: acute respiratory distress syndrome; ECMO: extracorporeal membrane oxygenation; VV: veno-venous

Study	Patient Characteristics	Type of Dissection	Time to ARDS	Management	Outcome
Litmathe and Dapunt [4]	Adult male	Traumatic Stanford type B dissection	Early after presentation	ECMO support	Survived
Xuan et al. [5]	Adult male	Uncomplicated Stanford type B dissection	Intraoperative	Mechanical ventilation and supportive care	Survived
Miyagawa et al. [6]	53-year-old male	Acute Stanford type B dissection	Day 4 after diagnosis	VV-ECMO and continuous renal replacement therapy	Survived

Acute aortic dissection is increasingly recognized as a systemic inflammatory disease rather than a purely structural vascular disorder. Disruption of the aortic wall initiates a complex inflammatory cascade involving leukocyte activation, endothelial dysfunction, oxidative stress, and release of pro-inflammatory cytokines. Elevated concentrations of inflammatory mediators, particularly IL-6 and CRP, have been associated with disease severity and adverse clinical outcomes in acute aortic syndromes [7-9]. This inflammatory response may extend beyond the aortic wall and contribute to distant organ dysfunction. At the pulmonary level, endothelial injury and increased alveolo-capillary permeability may promote non-cardiogenic pulmonary edema, acute lung injury, and, in rare circumstances, ARDS.

Several observations support an inflammation-mediated mechanism in our patient. First, respiratory deterioration occurred shortly after the diagnosis of acute aortic dissection. Second, inflammatory markers were markedly elevated, with a CRP concentration of 200 mg/L and leukocytosis of 15,000/mm³. Third, extensive investigations failed to identify cardiogenic, infectious, or thromboembolic explanations for the respiratory failure. Finally, subtle inflammatory pulmonary abnormalities were already visible on the admission CT scan before the subsequent development of diffuse bilateral infiltrates and severe hypoxemia. Taken together, these findings support the hypothesis that systemic inflammation triggered by the aortic injury may have played a central role in the development of ARDS. Nevertheless, a causal relationship cannot be definitively established from a single case report.

ESRD likely amplified this inflammatory process. Chronic kidney disease and long-term hemodialysis are associated with persistent low-grade inflammation, endothelial dysfunction, oxidative stress, immune dysregulation, and impaired clearance of inflammatory mediators [10,11]. Consequently, patients with advanced renal disease may exhibit exaggerated inflammatory responses when exposed to acute vascular insults. In the present case, chronic kidney disease may therefore have acted as a major susceptibility factor, facilitating progression from an initially uncomplicated aortic dissection to diffuse inflammatory lung injury. Furthermore, advanced age, neurological comorbidities, and limited physiological reserve likely reduced the patient's capacity to tolerate severe respiratory failure and contributed to the fatal outcome.

A major diagnostic challenge was distinguishing inflammatory ARDS from more common causes of respiratory deterioration in a patient with acute aortic dissection and chronic hemodialysis. Cardiogenic pulmonary edema was considered unlikely because serial electrocardiograms showed no ischemic changes, cardiac troponin levels remained negative, NT-proBNP levels showed no significant increase from baseline values, and repeat echocardiography demonstrated preserved left ventricular systolic function without evidence of elevated filling pressures. Pulmonary embolism had already been excluded by CTA, while infectious pneumonia was considered unlikely given the low procalcitonin level, negative microbiological investigations, and absence of convincing clinical evidence of infection despite marked inflammatory activation. The systematic exclusion of cardiogenic, embolic, and infectious etiologies considerably strengthened the diagnosis of inflammatory non-cardiogenic respiratory failure.

The diagnosis of ARDS was established according to the Berlin definition [3]. Respiratory symptoms developed within one week of a recognized clinical insult, namely acute Stanford type B aortic dissection. Bilateral pulmonary infiltrates were present on chest imaging (Figure 3), and cardiogenic pulmonary edema was excluded by repeat echocardiography demonstrating preserved left ventricular systolic function, stable NT-proBNP levels, and negative cardiac biomarkers. ABG analysis performed while the patient was receiving a fraction of inspired oxygen (FiO₂) of 1.0 revealed a PaO₂ of 67 mmHg, corresponding to a PaO₂/FiO₂ ratio of 67 mmHg and fulfilling the criteria for severe ARDS. Importantly, the admission CT scan demonstrated only a focal unilateral ground-glass opacity that did not satisfy imaging criteria for ARDS, suggesting that pulmonary involvement may initially be subtle before progressing rapidly toward diffuse inflammatory lung injury.

The role of TEVAR deserves specific discussion. Current guidelines recommend TEVAR for complicated type B dissections associated with rupture, impending rupture, malperfusion syndrome, refractory pain, uncontrolled hypertension, or progressive aortic enlargement [2]. None of these criteria were present at admission. Consequently, conservative medical management was considered the most appropriate initial strategy according to contemporary recommendations. Whether earlier endovascular intervention could influence the inflammatory cascade remains unknown and warrants further investigation.

This case also illustrates a challenging therapeutic dilemma. Optimal management of uncomplicated type B aortic dissection requires strict blood pressure and heart rate control to minimize aortic wall stress. In contrast, severe ARDS frequently necessitates aggressive ventilatory support, prone positioning, meticulous fluid management, and occasionally vasopressor therapy. Reconciling these potentially competing objectives may be particularly difficult in elderly patients undergoing chronic hemodialysis. The coexistence of these two severe conditions undoubtedly increased management complexity and may have contributed to the unfavorable outcome.

The potential role of anti-inflammatory therapy is also noteworthy. Recent evidence supports the use of corticosteroids in selected patients with moderate-to-severe ARDS, particularly when significant inflammatory activation is present [12,13]. Given the hypothesized inflammatory pathophysiology linking acute aortic dissection and ARDS, anti-inflammatory strategies may warrant consideration in future cases. However, the exceptionally rapid deterioration observed in our patient precludes any meaningful assessment of whether corticosteroids or other immunomodulatory therapies might have altered the clinical course.

From a practical perspective, clinicians should maintain a high index of suspicion for inflammatory pulmonary complications in patients with acute type B aortic dissection who develop unexplained dyspnea, increasing oxygen requirements, rising inflammatory markers despite the absence of infection, new pulmonary infiltrates on imaging, or worsening gas exchange without evidence of cardiogenic pulmonary edema. Recognition of these warning signs may facilitate earlier diagnosis, closer monitoring, and prompt multidisciplinary management.

This report has several limitations. As a single case report, it cannot establish a definitive causal relationship between acute type B aortic dissection and ARDS. Repeat thoracic CT imaging during respiratory deterioration could not be performed because of the patient's critical condition, necessitating follow-up with serial chest radiographs. Furthermore, although alternative etiologies were systematically investigated and largely excluded, occult contributing factors cannot be completely ruled out.

## Conclusions

ARDS represents a rare but potentially devastating complication of acute Stanford type B aortic dissection. This case illustrates how an initially uncomplicated dissection can rapidly evolve into severe non-cardiogenic respiratory failure and multiorgan dysfunction despite guideline-directed medical management. Although a causal relationship cannot be definitively established, the temporal association, marked inflammatory response, exclusion of alternative etiologies, and previously reported cases support the hypothesis of inflammation-mediated lung injury triggered by acute aortic dissection.

Advanced age, ESRD requiring chronic hemodialysis, and multiple comorbidities may have contributed to an exaggerated systemic inflammatory response and an unfavorable clinical outcome. Clinicians should therefore maintain a high index of suspicion when patients with acute type B aortic dissection develop unexplained dyspnea, increasing oxygen requirements, rising inflammatory markers, or new pulmonary infiltrates in the absence of cardiogenic, infectious, or thromboembolic causes. Greater awareness of this uncommon complication may facilitate earlier recognition, closer monitoring, and timely supportive management. Further studies are needed to better understand the mechanisms linking acute aortic dissection and ARDS and to identify patients at increased risk for inflammatory pulmonary complications.
